# Retinal Vaso-Occlusive Complications Associated with Systemic Lupus Erythematosus: A Systematic Review of Clinical Presentation and Visual Outcomes

**DOI:** 10.31138/mjr.120125.cas

**Published:** 2026-06-01

**Authors:** Zunaira Amjad, Fatima Ameer, Hareem Farooq, Hassan Farooq, Habibah Sheikh, Moeez Saqib, Muhammad Zain Ameer, Huzefa Habib, Muhammad Husnain Riaz, Amna Khalid, Aleenah Mohsin, Aqeeb Ur Rehman

**Affiliations:** 1 Department of Internal Medicine, Services Institute of Medical Sciences, Lahore, Pakistan;; 2 Department of Internal Medicine, Holy Family Hospital, Rawalpindi, Pakistan;; 3 Department of Internal Medicine, Mayo Hospital, Lahore, Pakistan;; 4 Department of Internal Medicine, Ameer-Ud-Din Medical, PGMI, Lahore, Pakistan;; 5 Department of Ophthalmology, Mayo Hospital, Lahore, Pakistan;; 6 Department of Internal Medicine, King Edward Medical University, Lahore, Pakistan

**Keywords:** Systematic Lupus Erythematosus, SLE, retinal vascular disease, rheumatology, ocular manifestations

## Abstract

**Background:**

Ophthalmic involvement in systemic lupus erythematosus (SLE), particularly retinal vascular diseases, remains under-recognised despite its potential to cause severe visual impairment. This systematic review aims to evaluate the clinical presentation, pathophysiology, and treatment outcomes of retinal vascular diseases associated with SLE.

**Methods:**

A comprehensive literature search was conducted across PubMed/MEDLINE, ScienceDirect, Cochrane and Google Scholar using a combination of relevant MeSH terms. Data were extracted independently and quality assessment was conducted using the Joanna Briggs Institute Critical Appraisal Tool.

**Results:**

A total of 35 studies, corresponding to 39 patients, were included in quantitative synthesis. Mean age of patients was 27.8±12.9 years. Most patients were from India (n=10; 25%) and the USA (n=8; 20%), with a majority being female (n=31; 79.5%). Unilateral visual disturbances were reported in 25 (64%) cases while 14 (36%) patients showed bilateral symptoms. Disease was sudden in onset in 23 (59%) and followed a progressive course in 16 (41%) patients. Common findings included Central Retinal Artery Occlusion (CRAO) n 24 patients (61.5%) and Central Retinal Vein Occlusion (CRVO) in 22 patients (56.4%). 36 patients (92%) were treated with steroids but only 25% responded positively, necessitating the use of immunosuppressants in 24 (61.5%) and anticoagulants in 16 (41%) patients. Among the 38 patients where outcomes were reported, only 2 (5%) achieved complete recovery, 14 (37%) showed partial recovery, and 20 (52.6%) showed no improvement.

**Conclusion:**

Retinal vascular involvement in SLE is rare but associated with significant visual morbidity. Early diagnosis and combined immunosuppressive therapy may improve outcomes. Further research is required to stan-dardise treatment protocols.

## INTRODUCTION

Systemic Lupus Erythematosus (SLE), a prototypic chronic autoimmune disease, is characterised by widespread inflammation and tissue damage due to an attack of the immune system on various organs.^1^ Affected organ systems may include skin, joints, kidneys, heart, gastrointestinal tract, lungs, eyes, and brain.^2^ The global incidence of SLE is estimated to be 5.14 per 100,000 person-years, with 0.40 million new cases annually.^1^ The disease is significantly more common in women than men, with a median onset age ranging from the late teens to early 40s.^3^ It is estimated that up to one-third of SLE patients experience some form of ophthalmic involvement ranging from relatively mild to severe, vision-threatening disease.^4^

Ocular manifestations of SLE are generally linked to active lupus or its associated comorbidities.^5^ Systemic lupus erythematosus (SLE) can affect virtually any part of the eye, such as the orbit, ocular adnexa, eyelids, choroid, sclera, cornea, retina, and optic nerve.^3^ Despite being more serious and vision-threatening, posterior eye vasculitis diseases receive significantly less attention as compared to anterior eye involvement due to their rarity compared to anterior eye diseases. The prevalence of lupus retinopathy ranges from 3% to 28%.^6^ Both retinal vasculitis and vaso-occlusive retinopathies, including central retinal artery occlusion (CRAO), branch retinal artery occlusion (BRAO), central retinal vein occlusion (CRVO), or branch retinal vein occlusion (BRVO), are caused by thromboses of the retinal arterioles due to deposition of immune complexes secondary to inflammation.^7^ While patients with mild retinopathy or those in early stages of retinopathy may not exhibit any obvious symptoms, severe and progressive retinopathy can result in visual distortions, visual field defects, and complete vision loss.^8^ The most frequently observed features of SLE retinopathy, such as cotton wool spots, retinal haemorrhages, and optic disk oedema, correlate with the activity of disease and hence may not be readily apparent.^9^

The large body of emerging literature on this topic necessitates an integrated write-up to better understand the clinical presentation, pathophysiology, prognosis, and outcome of this clinical association. An understanding of the disease process will allow timely diagnosis and improve prognosis by preventing the development of any late-stage complications including vision loss. This review serves to not only educate and guide practicing physicians, but also provides a foundation for researchers to build on this work as we expand the scientific understanding of this association. This is the first systematic review to provide a comprehensive outline of available evidence by incorporating case reports from multiple regions and healthcare settings, thus offering a global perspective on the ocular complications associated with SLE as well as their management options.

## METHODS

This article is fully compliant with the PRISMA (Preferred Reporting Items for Systematic Reviews) 2020 statement.^10^ Institutional ethics approval was not required for this retrospective data analysis. The review was registered on PROSPERO (CRD420251057175). The authors ensured that informed consent had been obtained from the patients for the original publication of their cases.

### Search strategy

A systematic literature search was conducted up to 1 January 2024 of the following four databases: PubMed/MEDLINE, Cochrane, ScienceDirect, and Google Scholar. The search string consisted of a combination of the following keywords and MeSH terms: “Lupus Erythematosus, Systemic” [MeSH], “SLE”, “Lupus Erythematosus”, “Retinal Artery Occlusion” [MeSH], “CRAO”, “BRAO”, “Retinal Vein Occlusion” [MeSH], “CRVO”, “BRVO”, “Retinal Vasculopathy”, and “Retinal vasculitis”.

On PubMed, the following query was used:

(“Lupus Erythematosus, Systemic”[MeSH] OR “SLE”[-Title/Abstract] OR “Lupus Erythematosus”[Title/Abstract])
AND
(“Retinal Artery Occlusion”[MeSH] OR “CRAO”[Title/Abstract] OR “BRAO”[Title/Abstract]
OR “Retinal Vein Occlusion”[MeSH] OR “CRVO”[Title/Abstract] OR “BRVO”[Title/Abstract]
OR “Retinal Vasculopathy”[Title/Abstract] OR “Retinal vasculitis”[Title/Abstract])

The complete search string used in each database is provided in the **Supplementary Item 1**. No filters regarding time, study design, language, or country of publication were used in the retrieval of the relevant publications.

### Eligibility criteria

All articles that described Retinal Vascular disease, confirmed by fundoscopic findings, in association with Systemic Lupus Erythematosus, were included. Articles describing patients in whom a diagnosis of Retinal vascular disease was not confirmed by fundoscopy, or in which a diagnosis was not associated with Systemic Lupus Erythematosus, were excluded.

### Study selection

**Figure 1.**
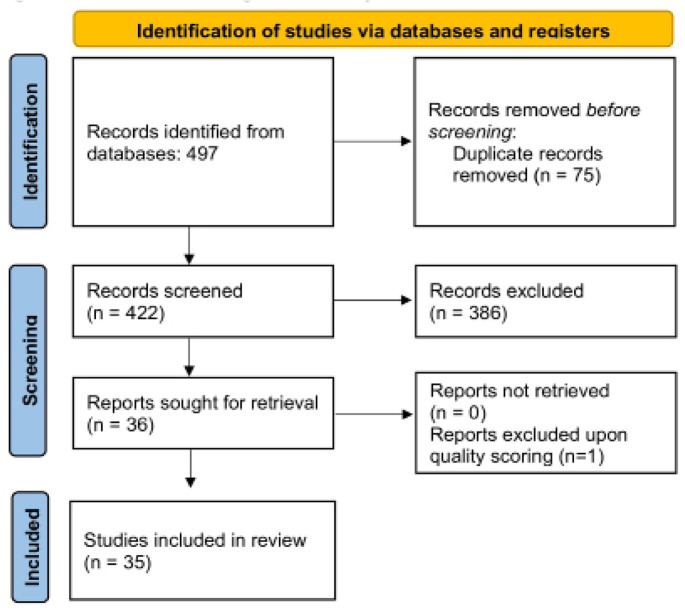
PRISMA flow diagram for study selection.

Relevant articles were selected and screened according to the PRISMA flowchart presented in **Figure 1**. The records identified through a preliminary search were downloaded into Mendeley (Elsevier, Amsterdam, Netherlands) and duplicates were removed. Two authors (MZA and FA) independently performed the screening and identified case reports, case series, abstracts, and letters to the editor reporting cases of Retinal vascular diseases in association with SLE. The bibliographies of these articles were also screened to identify any omitted cases.

### Quality assessment

The quality of the included articles was assessed by Joanna Briggs Institute Critical Appraisal Tool for case reports (JBI).^11^ Three reviewers first independently scored each article and then awarded a consensus score to each. The score report is provided in the **Supplementary Item 2**.

## RESULTS

Our search of four databases identified 497 articles. 75 articles were excluded due to duplication and 386 were removed due to irrelevance to the subject and 1 due to low quality score. The remaining 35 articles, including case reports, case series, and letters to the editor reporting cases of retinopathies in SLE were shortlisted after rigorous screening.^6,11–45^

The mean age of patients was 27.8±12.9 years (range 8-53 years). Highest number of patients were reported in India (n=10; 25%) and the USA (n=8; 20%). Out of 39 patients, 31 (79.5%) were females and 8 (20.5%) were males. 21 (53.8%) patients were known cases of SLE out of which 19 (90.47%) reported the time since onset (4.9±6.3 years). 18 (46.2%) patients had ophthalmic complications as the presenting manifestation of new onset SLE.

25 (64%) patients presented with unilateral visual disturbances while 14 (36%) patients showed bilateral symptoms. The onset of symptoms was sudden in 23 (59%) and slowly or rapidly progressive in 16 (41%) of the patients. SLE predominantly presented along with other manifestations, such as dermatologic manifestations (n=22; 56%), blood cell defects (n=16; 41%), arthritis (n=15; 38.5%), and lupus nephritis (n=7; 18%). CRAO, CRVO, BRAO, BRVO, and vasculitis were often seen coexisting pathologically with CRAO being the most predominant one affecting 24 (61.5%) patients, closely followed by CRVO seen in 22 (56.4%) patients. Additionally, BRAO was reported in 5 (12.8%), vasculitis in 3 (7.7%) and BRVO in 1 (2.5%) patient. 43.5% of the patients (n=17) also tested positive for anti-phospholipid antibody.

Management primarily consisted of steroids (prednisone, prednisolone; n=36; 92%). However, only 25% of patients responded positively (completely or partially) to steroid-only treatment and hence required immuno-suppressants (n=24; 61.5%), anticoagulants (n=16; 41%), and aspirin (n=6; 15%). The major immunosuppressants used were hydroxychloroquine (n=14), cyclophosphamide (n=7) and azathioprine (n=7). Of the 38 patients for whom outcomes were reported, two (5%) achieved complete recovery while 14 (37%) recovered partially. Twenty (52.6%) patients showed no improvement at all, and two (5%) patients reported further worsening of vision on follow-up.

## DISCUSSION

Systemic Lupus Erythematosus (SLE) is a multisystem autoimmune disease with remarkably heterogeneous clinical features. Virtually any organ system can be affected, including kidneys, skin, heart, lungs, brain, blood vessels, and the eyes.^46^ Although the ocular system is typically seen as distinct from the immune system, the systemic inflammation caused by SLE can affect eye structures, with an estimated one-third of SLE patients exhibiting eye involvement.^47,48^ In SLE, retinal involvement—manifesting as haemorrhages, cotton-wool spots, retinal oedema, and vascular occlusions—occurs in about 10% to 29% of patients. By contrast, retinal vasculitis and optic neuritis are uncommon but serious complications that can cause marked and often irreversible vision loss if not promptly treated.^49^ The goal of this systematic review is to evaluate all existing literature on retinal vascular diseases in patients with systemic lupus erythematosus (SLE) and update the current understanding of the clinical and diagnostic aspects of vasculitic and non-vasculitic retinal manifestations in this widespread autoimmune disorder. While literature shows a 90% preponderance of SLE towards females, interestingly our systematic review indicates that eye involvement with SLE has a comparatively lower female-to-male ratio of approximately 3:1. SLE usually presents with joint pain as well as a multitude of dermatological manifestations such as dermatitis, malar rash, or alopecia.^50^ Our systematic review also demonstrated coexisting dermatological findings (56%) as the most common presentation of SLE, closely followed by blood cell defects (41%) and arthritis (38.5%). SLE can involve any layer of the eye and hence ocular manifestations vary significantly and have been seen to include abnormalities of eyelids, choroid, sclera, cornea, retina, and optic nerve.^51^ Retinal involvement is estimated to occur in 3–28% of known SLE cases and is the most common cause of vision loss in SLE patients.^52^

Our systematic review focuses solely on the involvement of the posterior segment of the eye in SLE patients and encompasses disorders including, but not limited to, vasculitis, vaso-occlusion, and retinopathy. With the exception of retinal vaso-occlusive disease, the prevalence of each of the other ocular manifestations has been consistently below 5% in literature previously.^53^

Similar findings were seen in our review with only 17.5% of the cases involving vasculitis, retinopathy and microangiopathy combined. The co-occurrence of both branch retinal artery occlusion (BRAO) and central retinal artery occlusion (CRAO) along with central retinal vein occlusion (CRVO) in patients with systemic lupus erythematosus (SLE) is extremely rare;^11^ another finding that is corroborated by our results.

The pathogenesis of SLE is intricate and multifaceted, involving a combination of genetic, epigenetic, immunoregulatory, and environmental factors that influence susceptibility, onset, progression, and prognosis in each patient. Inflammation in lupus is primarily caused by the formation of autoantibodies and immune complexes, which initiate inflammatory responses and activate the complement system,^52^ which in turn can attack virtually any organ system. Although the exact pathogenesis of retinal vascular defects is understudied and hence still unknown, one theory suggests that immune dysregulation in SLE drives disease flares through elevated levels of both innate cytokines (such as interleukin-1a and type I interferons (IFNs)) and adaptive cytokines (including Th1, Th2, and Th17 types). This dysregulation also involves increased production of IFN-associated chemokines and soluble members of the tumour necrosis factor (TNF) superfamily resulting in hypercoagulability, and hence retinal vaso-occlusion.^11^ Immunohistochemical studies in patients with retinal vasculitis disclose immune complex deposition within the vessel walls, which may also independently lead to vaso-occlusion in the eye.^54^ This immune complex deposition can set off a cascade that leads to fibrinoid degeneration of the retinal vessel walls, giving rise to the vascular defects pathognomonic of SLE-associated retinal vascular diseases.^9^

**Table 1.** Demographic and clinical characteristics of patients with retinal vascular diseases associated with SLE.

**Sr No.**	**Author/Year**	**Age, Sex**	**Onset of SLE**	**Ocular Symptoms**	**Clinical manifestations of SLE**	**VA** *(at presentation)*	**Fundoscopic findings**	**Fluorescein angiogram**	**APS**	**Diagnosis**	**Treatment**	**Outcome**
1	Akhlagi et al. 2017^12^	29,F	Known case for 18 years	Bilateral rapidly progressive visual loss	Arthritis, Haemolytic anaemia, Pleuritis, ANA (+), Anti-sm (+)	**(OD)** NPL; **(OS)** NPL	**(OU)** Vitreous haemorrhage and widespread superficial and deep intraretinal haemorrhages	Delay in filling of choroidal and retinal vessels, filling within 25 seconds after dye injection	No	CRAO, BRAO, CRVO	IV heparin, methylprednisolone, azathioprine, prednisolone	Vision failed to improve significantly
2	Bawankar et al. 2018^13^	14,F	New onset	Sudden loss of vision in left eye	Butterfly rash, Pancytopenia, Lupus nephritis, ANA (+), Anti-dsDNA (+)	**(OD)** 6/6; **(OS)** HM	**(OS)** Disc oedema, pale and oedematous retina, engorged and tortuous retinal veins, scattered intraretinal haemorrhages in all quadrants and a cherry-red macular spot	n/r	No	CRAO, CRVO	n/r	Vision failed to improve significantly. Death due to pneumonitis
3	Boudoux et al. 2020^14^	44,F	Known case	Acute blurred vision in the left eye	Arthritis, Pericarditis	**(OD)** 6/6; **(OS)** 6/6	**(OS)** Haemorrhagic occlusive retinal vasculitis in supertemporal arcade with vitreous haze	**(OS)** supertemporal occlusive retinal vasculopathy with nonperfusion areas	n/r	Retinal vasculitis	IV methylprednisolone, PPV, Mycophenolate mofetil	Complete recovery
4	Brahim et al. 2022^6^	40,M	New onset	Recurrent blurred vision in the right eye	Malar rash, ANA (+)	**(OD)** 6/21; **(OS)** 6/6	**(OD)** Superficial flame-shaped retinal haemorrhages, and macular oedema	**(OD)** Vascular tortuosity, retinal haemorrhage, and cotton wool spots.	Yes	CRVO, BRVO	HCQ, intra-vitreal anti-VEGF, aspirin	Partial recovery
5	Chandran et al. 2020^15^	22,F	Known case for 6 months	Bilateral sudden, painless decrease of vision.	Malar rash, oral ulcers, nephritis, ANA (+), Anti-dsDNA (+), Anti-Sm (+)	**(OD)** CF; **(OS)** CF	Pallor of the optic disc **(OS > OD)**with multiple peripapillary cotton-wool spots **(OD > OS)**. Numerous dot and blot haemorrhages in the posterior pole and midperiphery. Hard exudates in clusters just inferior to the fovea **(OD)**. Diffuse retinal whitening along with cherry-red spot at the macula	Delayed arm to choroid circulation time, delayed arterial filling (**OD** 38 s and **OS** 42 s), delayed arteriovenous transit circulation and extensive capillary non-perfusion areas with no neovascularisation in both eyes.	No	CRAO	0.5% Timolol eye drops along with ocular massage. Aspirin 75mg, PRP	No improvement
6	Chang et al. 2010^16^	35,F	Known case for 7 years	Sudden loss of vision in the right eye	Pancytopenia, non-erosive arthritis. ANA (+), Anti-dsDNA (+)	**(OD)** 6/60; **(OS)** 6/6	**(OD)** Pale and oedematous retina, engorged and tortuous retinal veins, a few flame-shaped haemorrhages and a mild swollen disc.	**(OD)** delayed choroidal and arteriolar filling, prolonged arteriovenous transit time (14s) and mild late staining of the disc.	Yes	CRAO, CRVO	Methylprednisolone, subcutaneous LMWH	Rapid improvement in vision over 2 weeks
7	Durukan et al. 2005^17^	23,F	Known case for 3 years	Sudden loss of vision in the right eye	Abortions, Arthritis, Malar rashes, Haemolytic anaemia, ANA (+), Anti-dsDNA (+)	**(OD)** NPL; **(OS)** 6/6	**(OD)** Venous stasis retinopathy combined with CRAO. Retina pale and oedematous, with widespread flame and intraretinal dot haemorrhages in the four main quadrants, swollen optic disc, arterial sheathing, engorged and tortuous retinal veins, and cherry-red spot	**(OD)** Disc oedema and delayed arteriolar filling, with congested, dilated veins that did not fill with fluorescein.	Yes	CRAO, CRVO	Subcutaneous Heparin, Prednisolone, Pentoxifylline and ASA.	No improvement. Vision NPL
8	Guleria et al. 2020^18^	9,M	New onset	Decreased vision bilaterally	Pancytopenia, neurological symptoms, ANA (+)	**(OD)** LP; **(OS)** LP	**(OU)** Cotton-wool spots with few retinal haemorrhages. Thinned out and narrow arterioles showing the presence of tram-tracking. Characteristic beaded appearance of retinal venules.	**(OU)** Significant central non-perfusion of the retina	No	Retinal vasculopathy	Methylprednisolone, oral prednisolone, warfarin, aspirin, HCQ, Cyclophosphamide.	Vision improved gradually to a visual acuity of 6/36 in both eyes
9	Guleria et al. 2021^18^	8,M	New onset	Decreased vision bilaterally	Malar rash, Mucosal ulcers, Anaemia, Lymphopenia, Nephritis, ANA (+), Anti-dsDNA (+)	n/r	**(OU)** Pre-retinal peri-papillary haemorrhages, multiple cotton-wool spots and venous congestion	**(OU)** Blocked fluorescence due to the haemorrhage and leakage of the dye.	No	Microangiopathy	Cyclophosphamide, Methylprednisolone (30 mg/kg/day), oral prednisolone and HCQ.	Normal vision at 9 months follow-up
10	Hamano et al. 2001^19^	17,F	New onset	Decreased vision in left eye	Fever, malar rash, polyarthralgia, oral ulceration, pleuritis, ANA (+), Anti-dsDNA (+)	**(OD)**6/6; **(OS)** LP	**(OS)** Diffuse haemorrhages involving the nasal half of the macula as well as macular oedema	**(OS)** Extensive areas of retinal nonperfusion and widespread leakage from the retinal veins	No	CRVO	Aggressive immunosuppressive therapy (steroids, warfarin, plasma exchange)	Unchanged visual acuity and retinal findings on 100 days follow-up
11	Hong-Kee et al. 2014^20^	19,F	Known case for 3 years	Sudden painless loss of vision in Left eye	n/r	**(OD)** 6/6; **(OS)** HM	**(OS)** Extensive flame shaped haemorrhages, dilated tortuous veins, swollen hyperaemic disc.	**(OS)** Delayed venous filling, prolonged arteriovenous transit time with extensive capillary non perfusion affection 360 degree of peripheral retina with macular ischemia	Yes	CRVO	IV methylprednisolone followed by oral prednisolone, HCQ, warfarin, PRP	Partial improvement
12	Hong-Kee et al. 2014^20^	16,F	Known case for 10 months	Bilateral Central Scotomas	Lupus Nephritis	**(OD)** 6/24; **(OS)** 6/24	**(OU)** Multiple cotton wool spots, intra retinal haemorrhage, macular oedema	Perifoveal vasculitis with macular ischemia and capillary nonperfusion at superior, temporal, and inferior parts of the retina in both eyes **(OS>OD)**	Yes	Vaso-occlusive lupus retinopathy	IV methylprednisolone followed by oral prednisolone, HCQ, warfarin, PRP	Partial improvement
13	Hua et al. 2015^21^	20,F	New onset	Sudden bilateral vision loss	Malar rash, ANA (+), Anti-Sm (+)	**(OD)** CF; **(OS)** CF	**(OU)** Pale oedematous retina with cherry red macula.	**(OU)** Delay in arterial filling and delayed arterial venous transit consistent with CRAO	No	CRAO	Heparin, Prednisone, HCQ, AZP	Minimal improvement
14	Huang et al. 2020^22^	11,F	New onset	Sudden painless vision loss in left eye	Lupus nephritis, ANA (+), Anti-dsDNA (+)	**(OD)** 6/6; **(OS)** HM	**(OS)** CRAO, CRVO, diffuse retinal haemorrhages, tortuous dilatation of vessels, optic disc oedema	n/r	Yes	CRAO, CRVO	HCQ, IV methylprednisolone, PRP	Slight improvement
15	Hwang et al. 2012^23^	18,F	New onset	Sudden marked visual loss in right eye	Fever, malar rashes, pleuritis, nephritis, ANA (+), Anti-dsDNA (+)	**(OD)** HM; **(OS)** 6/6	**(OD)** Pale and oedematous retina with widespread flame haemorrhages, swollen optic disc, engorged and tortuous retinal veins and cherry red spot	**(OD)** Disc oedema and delayed arteriolar filling, dilated veins that did not fill with fluorescein	Yes	CRAO, CRVO	Steroids, anticoagulants, PRP	No improvement
16	Ish et al. 2020^24^	52,M	New onset	Sudden painless loss of vision in left followed by right eye	Facial rash, oral ulcers, anaemia, photophobia, ANA (+)	**(OD)** NPL; **(OS)** NPLL	**(OD)** Pale disc and retina with cherry red spot at fovea with arteriolar attenuation. (**OS)** Disc oedematous and pale, whole retina pale with attenuation of both veins and arteries	n/r	No	CRAO	Immediate ocular massage, IV mannitol, IV methylprednisolone	No improvement
17	Ismail et al. 2022^25^	42,F	New Onset	Sudden, profound, painless decrease of vision in left eye	Thrombocytopenia, alopecia, ANA (+)	**(OD)** 6/6; **(OS)** HM	**(OS)** Cherry red spot appearance and the surrounding whitening of the macular area and the corresponding widening of fovea	**(OS) cherry red spot appearance and the surrounding whitening of the macular area and corresponding widening of foveal avascular zone**	Yes	CRAO	HCQ, Oral steroids	Patient developed HCQ maculopathy with no improvement in vision
18	Silverman et al. 1978^26^	15,M	New onset	Conjunctival infection in both eyes and sub conjunctival haemorrhage in the right	Polyarthralgias, Malar rash, Oral ulcers, ANA (+), Anti-dsDNA (+)	**(OD)** 6/6; **(OS)** PL	**(OS) R**etinal haemorrhages, disc totally obscured by haemorrhage, marked venous distention, retinal arterioles slightly narrowed, and diminished arteriolar light reflex	n/r	n/r	CRVO, CRAO	Prednisone 60mg/day	No improvement
19	Laroche et al. 1984^27^	53,F	Known case for 13 years	Visual haze	n/r	**(OD)** 6/6; **(OS)** 6/7.5	**(OS)** papilledema, venous tortuosity, engorgement and sheathing, many small diffuse intraretinal haemorrhages, few cotton wool spots	**(OS)** marked venous filling delay. Disc and perimacular capillaries swollen; few scattered areas of obliterated capillary bed, late phase fluorescein angiograms disclosed a huge dye leakage from the veins in areas with bright parietal staining	n/r	CRVO	Prednisone 20mg/day, heparin	Partial improvement
20	Joshi et al. 2018^28^	23,F	Known Case	Sudden painless vision loss in right eye	Pericarditis, ANA (+), Anti-dsDNA (+), Anti-Sm (+)	**(OD)** NPL; **(OS)** 6/6	**(OD):** diffuse macular oedema with central cherry red spot and boxcarring of blood in arterioles consistent with CRAO	n/r	Yes	CRAO	Methyl prednisolone, Plasmapheresis, Azathioprine, HCQ, Warfarin	Partial improvement
21	Khan et al. 2015^29^	21,F	New onset	Progressive worsening of vision in right eye	Embolic stroke, Libman-Sacks endocarditis, ANA (+), Anti-Sm (+)	**(OD)** VFD in all quadrants. **(OS)** Inferior VFD	**(OU)** CRAO and vitreous haemorrhage	n/r	Yes	CRAO	IV heparin with oral warfarin, Oral prednisolone	No improvement
22	Korematsu et al. 2014^30^	15,F	Known case for 1 year	Sudden decrease of vision in left eye	Butterfly rash and photosensitivity, ANA (+)	**(OD)** 6/5; **(OS)** 6/120	**(OS)** Massive intraretinal haemorrhage due to CRVO	n/r	Yes	CRVO	Intravenous LMWH, steroids, oral fexofenadine and levocabastine eye-drops	Partial improvement
23	Kumar et al. 2021^31^	44,F	Known case for 10 years	Bilateral decrease of vision	Haemolytic anaemia, leukopenia, non-erosive arthritis, pleural effusion, lupus nephritis, Malar rash, ANA (+), Anti-dsDNA (+)	**(OD)** 6/60; **(OS)** 6/120	**(OD)** Diffuse arteriolar narrowing, dot-blot haemorrhages, cotton wool spots. **(OS)** sclerosed super-temporal artery with cattle-tracking of blood in multiple arterioles at the posterior pole, diffuse cotton wool spots, and retinal pallor corresponding to BRAO	**(OS)** multiple arteriolar affections in the early phase with pruned vascular appearance, arteriolar leakage in the late phase, capillary drop-outs, capillary non-perfusion areas, and non-filling of temporal retinal arterioles	No	BRAO	IV methylprednisolone, IV rituximab, Oral prednisolone, HCQ, Azathioprine	BCVA improved
24	Kumar et al. 2021^31^	51,F	Known case for 21 years	Decrease of vision in right eye	Non-erosive arthritis, anaemia, thrombocytopenia, Malar rash, fever, ANA (+), Anti-dsDNA (+)	**(OD)** 6/12; **(OS)** 6/6	**(OD)** Dot-blot haemorrhages, dilated tortuous veins, temporal peripapillary, and inferior macular retinal pallor with narrowing and cattle-tracking signs in the corresponding arterioles	**(OD)** Delayed filling of the branch retinal artery in the region of the pallor, delayed filling of veins with increased arteriovenous passage time of 28 s, and inflammatory vascular staining	Yes	Combined infero-temporal BRAO with non-ischemic CRVO (NICRVO)	IV Methylprednisolone, subcutaneous LMWH followed by oral prednisolone in a tapering fashion	No improvement
25	Kumar et al. 2021^31^	34,M	Known case for 4 years	Sudden decrease of vision in left eye	Non-erosive arthritis, Lupus nephritis, Haemolytic anaemia, ANA (+), Anti-dsDNA (+)	**(OD)** 6/6; **(OS)** PL	**(OS)** Dot-blot haemorrhages, inferotemporal pallor along with narrowing and cattle-tracking of blood in the branch retinal artery, dilated tortuous veins, and disc hyperaemia with oedema	**(OS)** Slow filling of the infero-temporal branch retinal artery, increased AV passage time of 23 seconds, and diffuse vascular staining	No	BRAO with NICRVO	IV Methylprednisolone	No improvement
26	Leibovitch et al. 2001^32^	23,M	Known case for 6 years	Sudden unilateral decrease of vision in right Eye	Oral ulcers, Lupus Nephritis and renal failure, thrombocytopenia	**(OD)** NPL; **(OS)** 6/6	**(OD)** Swollen optic disc, arterial sheathing, scattered intraretinal haemorrhages in four quadrants and a cherry-red macular spot.	n/r	No	CRAO, CRVO	IV Steroids	No Improvement.
27	Lim et al. 2023^33^	41,F	Known case for 3 years	Sudden unilateral decrease of vision right eye	Leukopenia, thrombocytopenia, arthralgia, ANA (+), Anti-Sm (+)	**(OD)** NPL; **(OS)** 6/7	**(OD)** optic disc oedema and diffuse pallor with a cherry red spot	**(OD)** Severe delay and limitation in arterial filling	Yes	CRAO	Steroids	n/r
28	Mendrinos et al. 2008^34^	42,F	Known case for 6 months	Sudden bilateral loss of vision with no perception of light	Photosensitivity, Rash, Alopecia, polyarthritis, Autoimmune Haemolytic Anaemia, Neutropenia, Thrombocytopenia, ANA (+), Anti-dsDNA (+)	**(OD)** NPL; **(OS)** NPL	**(OU)** Pale optic discs and extensive and severe arteriolar narrowing. Superficial and deep intraretinal haemorrhages were scattered throughout the retina	**(OU)** Widespread retinal capillary non-perfusion with dye arrest at about one to two disc diameters away from the disc	No	CRAO, CRVO	IV followed by oral prednisone and cyclophosphamide.	No improvement.
29	Narang et al. 2021^35^	19,F	New onset	Sudden bilateral decrease of vision	Discoid rash, ANA (+), Anti-dsDNA (+)	**(OD)** HM; **(OS)** LP	**(OD)** Pale oedematous retinas with sparing of the cilioretinal artery area. Arterial attenuation with perivascular retinal haemorrhages. **(OS)** Similar findings in the superior half of retina	n/r	No	Combined CRVO in the right eye and combined BRVO in the left eye	Oral prednisolone, cyclophosphamide IV	No improvement in Right eye; slight improvement in Left eye
30	Noma et al. 2013^36^	33,F	Known case for 6 months	Sudden left sided decrease of vision	Malar rash, Polyarthritis, Leukopenia, ANA (+), Anti-dsDNA (+)	**(OD)** 6/6; **(OS)** 6/36	**(OS)** Superficial and deep intraretinal haemorrhages scattered throughout the retina.	**(OS)** CME without ischemia of the macular region or peripheral retina	No	CRVO	2 intra-vitreous injections of bevacizumab	Vision improved
31	Nishiguchi et al. 2013^37^	33,F	Known case for 3 months	Sudden loss of vision in left followed by right eye	Discoid rashes, Oral ulcers, pancytopenia, ANA (+), Anti-dsDNA (+)	**(OD)** 6/6 progressed to NPL; **(OS)** CF progressed to NPL	**(OU)** Swollen pale retina, tortuous vessels, wedge shaped haemorrhages, cherry red spot,	**(OU)** Delay in filling of choroidal retinal vessels ∼27 seconds after the dye	Yes	CRAO, Retinal vasculitis	IV methylprednisolone followed by oral prednisolone.	No improvement
32	Páramo et al. 2018^38^	14,M	New onset	Sudden visual loss in the Left eye	Pleuritis, Leukocytosis, thrombocytopenia, proteinuria	**(OD)** 6/6; **(OS)** LP	**(OS)** Disc oedema, generalised vascular dilatation and tortuosity, flame and dot haemorrhages in the 4 quadrants and generalised retinal pallor.	**(OS)** Delay in arterial filling and generalised hypofluorescence due to filling defect were observed	Yes	CRAO, CRVO	Steroids, Systemic anticoagulants, Vitrectomy	No improvement
33	Parchand et al. 2016^39^	16,F	Known case for 6 months	Sudden loss of vision in Left eye.	Hair loss, Arthritis, Oral ulcers, Leukopenia, ANA (+)	**(OD)** 6/6; **(OS)** LP	**(OS)** Disc oedema, dilated tortuous thrombosed retinal vein, arteriolar attenuation, flame shaped haemorrhages, pale retina, cherry red spot	n/r	No	CRAO, CRVO	Steroids, anticoagulants, methotrexate, PRP	No improvement.
34	Radosavljević et al. 2016^40^	36,F	Known case for 6 months	Sudden loss of vision in Right eye	Leukopenia, thrombocytopenia, ANA (+)	**(OD)** 6/12; **(OS)** 6/18	**(OU)** Perivascular sheathing, cotton wool spots, intraretinal haemorrhages, retinal oedema, wide areas of vascular occlusion.	**(OU)** Bilateral complete obliteration of macular capillaries and widespread occlusion of retinal vessels.	No	CRAO, CRVO	IV followed by oral prednisone and PRP	First improved, then worsened
35	Saraf et al. 2015^41^	22,F	New onset	Progressive vision loss	Hair loss, Skin changes, anaemia, thrombocytopenia, nephritis, ANA (+), Anti-Sm (+)	**(OD)** HM; **(OS)** 6/60	**(OU)** Pale optic nerves, disc haemorrhages, disc neovascularisation. arterial attenuation, cherry red spot bilateral, arterial boxcarring, multiple retinal haemorrhages, retinal whitening.	**(OU)** Bilateral extensive retinal non perfusion	Yes	CRAO	IV methylprednisolone followed by oral prednisolone, IV cyclophosphamide	Improved VA after 7 months
36	Song et al. 2001^42^	18,F	New onset	Acute bilateral decrease in visual acuity	Rash, ANA (+)	**(OD)** 6/60; **(OS)** CF	**(OU)** Retinal arteries constricted and veins surrounded by exudates. **(OS)** Profuse vitreous and disc haemorrhage and ghost vessels.	**(OU)** Ischemic changes secondary to non-perfusion and leakage of dye from retinal vessels	No	Retinal Vasculopathy	Laser photocoagulation, Methylprednisolone, oral prednisone, hydroxychloroquine	Vision improved
37	Storey et al. 2017^43^	42,F	Known case for 6 months	Acute bilateral vision loss	Arthritis, Rash, pancytopenia ANA (+), Anti-dsDNA (+), Anti-Sm (+)	**(OD)** 6/60; **(OS)** 6/120	**(OU)** Fundus examination revealed diffuse, confluent cotton-wool spots, and multiple ghost vessels.	**(OU)** Bilateral venous and arterial filling defects and severe vascular leakage in both eyes	Yes	RAO, RVO, Vasculitis	Corticosteroids, hydroxychloroquine, cyclophosphamide, bilateral PRP	BCVA improved.
38	Zhang et al. 2022^44^	31,F	New onset	Sudden loss of vision in Left eye	Rash, Hair loss, Anti-dsDNA (+), Anti-Sm (+)	**(OD)** 6/6; **(OS)** 6/15	**(OS)** Macular oedema, gray-white ischemia, cotton wool spots, arterial attenuation near macula, branch retinal veins slightly tortuous and dilated.	**(OS)** multiple ischemic areas in the area around macula, arteriolar attenuation, and nonperfusion arteries in temporal and superior temporal lobe.	No	BRAO	IV methylprednisolone	Vision improved.
39	Zou et al. 2012^45^	42,F	New Onset	Sudden decrease of vision in Left followed by right eye	Hair loss, Arthritis, ANA (+)	**(OD)** HM; **(OS)** HM	*On day 1*,**(OS)** Pale retina with cherry red macula in left eye. **(OD)** normal; *On day 18,* **(OS)** Pale optic disc, narrow arteries. **(OD)** Severe arteriolar narrowing, pale retina, cherry red macula.	*Day 1,* **(OS)** retinal ischemia, slow perfusion of retinal artery and vein in early phase; *On day 18*, delayed venous and arterial filling with obliteration of macular capillary, hypofluorescence on optic disk **(OD)**. Retinal circulation in left eye was relatively normal	No	CRAO	Oral prednisone, Cyclophosphamide	No improvement

APS: Antiphospholipid antibody syndrome; SLE: Systemic Lupus Erythematosus; VA: Visual Acuity; PR: Pupillary reaction; BCVA: Best corrected Visual acuity; OU: Both Eyes; OD: Right Eye; OS: Left Eye; CRAO: Central Retinal Artery Occlusion: BRAO: Branch Retinal Artery Occlusion: CRVO: Central Retinal Vein Occlusion: BRVO: Branch Retinal Vein occlusion; CF: Counting Fingers; HM: Hand Movement; LP: Only Light perception present; NPL: No Perception of light; RAPD: Relative afferent pupillary defect; VFD: Visual Field defect; ANA: Anti-nuclear Antibodies; CRP: C- Reactive Protein; ESR: Erythrocyte Sedimentation Rate; PRP: Panretinal Photocoagulation; PPV: Pars Plana Vitrectomy; HCQ: Hydroxychloroquine; n/r: not reported.

It has also been documented that patients with SLE who test positive for antiphospholipid antibodies (aPL) are more susceptible to recurrent retinopathy episodes, including arterial and venous thrombosis, optic disc oedema, and haemorrhages. ^55^ In our review, although all patients experienced retinal vascular events, recurrence data was not consistently reported, limiting the ability to assess any association between aPL positivity and event recurrence. While the design of this study inherently does not prove causation between the two, these findings are important for both, researchers to design studies to investigate this association (if any), and primary care physicians, rheumatologists, and ophthalmologists to be aware of this likely association and appreciate the need for regular ophthalmologic evaluations in SLE patients with aPL and emphasise the importance of testing for aPL in patients presenting with a suggestive clinical picture. Moreover, Bashiri et al. reported a 15.8% prevalence of retinopathy in newly diagnosed yet asymptomatic SLE patients- an unexpectedly high figure that reinforces the need to include ocular evaluation as a standard screening component at the time of SLE diagnosis.^56^

The diverse nature and multisystem involvement of SLE makes management particularly challenging. The primary objective of therapy is to achieve and sustain disease remission and prevent relapses.^52^ Recent optical coherence tomography angiography (OCTA) studies have shown that even SLE patients without clinical signs of ocular disease demonstrate rarefaction of the retinal microvasculature, with measurable reductions in vessel diameter, length, and fractal dimension, findings that support the presence of subclinical small-vessel involvement in lupus.^57^ Given the poor prognosis of ophthalmic complications even with aggressive treatment, early prevention offers the best chance of preserving vision.^47^ Corticosteroid therapy alone is generally insufficient for systemic disease control, frequently resulting in recurrence.^48^ In our review, only 25% of patients benefited from steroids alone. Consequently, it is recommended to administer immunosuppressants alongside steroids to reduce the likelihood of flares.^48^ However, even with combined immunosuppressants and steroids, only 54% of patients in our quantitative synthesis showed partial or complete recovery, indicating the need for better treatment options as well as preventive management. A potential treatment strategy could involve using methylprednisolone for its rapid immunosuppressive effects and rituximab for its long-lasting therapeutic benefits, combined with intravitreal anti-VEGF and photocoagulation therapy.^13^ There is a general lack of therapeutic consensus to help guide therapeutic decisions in lupus retinal defects; information is based mainly on case reports and small case series, and is sometimes extrapolated from the management of other lupus manifestations and other vasculitides.^58^

To the best of our knowledge, this is the first systematic review to comprehensively assemble evidence on the presentation, diagnosis, and treatment of retinal vascular diseases in association with SLE. Several reviews have explored ocular manifestations of SLE more broadly, including narrative and systematic reviews such as those by Palejwala et al. and Stafford-Brady et al., which offer useful context despite not focusing specifically on retinal vascular involvement.^3, 59^ This review builds upon their findings, adding quantitative synthesis to this neglected SLE presentation. To our knowledge, no prior publication has comprehensively synthesised case-level data focused specifically on retinal vaso-occlusive complications of SLE. An awareness of such an association may aid in early diagnosis, reduction of morbidity, and a better prognosis for patients of SLE. We would also like to acknowledge a few limitations in our findings. Firstly, the results are subject to inherent variability due to differences among individual patients and the subjective nature of case reporting by physicians. Secondly, publication bias raises concerns about the applicability of our findings to the general population. Despite these issues, the thorough and structured approach to literature searching and data extraction employed in this review ensures a comprehensive and reliable overview of the current understanding of the clinical and pathophysiological aspects of ocular manifestations of SLE.

## CONCLUSION

The association between systemic lupus erythematosus (SLE) and retinal vascular disease underscores the need for better clinical awareness. Given the poor prognosis of ophthalmic complications, focus on preventive measures and early intervention is essential to preserving vision in affected patients. Further research and standardised treatment protocols are necessary to improve outcomes for those with SLE-related ocular manifestations.

## Data Availability

All relevant data are available in the published manuscript or its supplements.

## AI AND EDITING SUPPORT

No commercial editing services or artificial intelligence were used in the preparation of this manuscript .
